# Enorme kyste hydatique cérébral révélé par un coma

**Published:** 2012-11-23

**Authors:** Khalid Khattala, Aziz Elmadi, Mohamed Rami, Abdelhalim Mahmoudi, Youssef Bouabdallah

**Affiliations:** 1 Service de chirurgie pédiatrique, CHU Hassan II, Fès, Maroc

**Keywords:** kyste hydatique, kyste cérébral, hydatid cyst, cerebral cyst

## Abstract

La localisation cérébrale du kyste hydatique est rare (2%), nous rapportons le cas d'une fille de 6 ans, admise aux urgences pour un coma d'installation progressive et dont le bilan radiologique a montré un énorme kyste cérébral fronto-pariétal, le diagnostic de kyste hydatique a été retenu après traitement chirurgical. C'est le premier cas du kyste hydatique, à notre connaissance, qui a été révélé par un coma, la malade a bien évolué après le traitement chirurgicale sans récidive après deux ans de recul.

## Introduction

La maladie hydatique est une affection parasitaire secondaire à l'infestation de l'organisme par l'embryon hexacanthe d'Echinococcusgranulosus [1, 2]. Au Maroc, l'hydatidose sévit à l'état endémique [3]. L'organe le plus touché est le foie, la localisation cérébrale est rare et n'excède pas 2%, elle touche essentiellement l'enfant [1]. Nous rapportons le cas d'une observation exceptionnelle d'un énorme kyste hydatique cérébral révélé par un coma et qui a bien évolué après traitement chirurgical.

## Patient et observation

F.L fille âgée de 6 ans, unique de sa fratrie, habitant le milieu rural avec notion de contact avec les chiens, le développement psychomoteur et affectif est normal. Elle a présenté des céphalées chroniques évoluant depuis 3 ans avec une macrocranie d'aggravation progressive, sans vomissements ni crises convulsives ni déficit moteur.

Au terme de ces 3 années, elle a présenté de façon rapidement progressive des céphalées intenses, des vomissements, une paraparésie puis des troubles de la conscience, ce qui a motivé une consultation aux urgences chirurgicales pédiatriques trois jours après le début des troubles.

**Figure 1:**
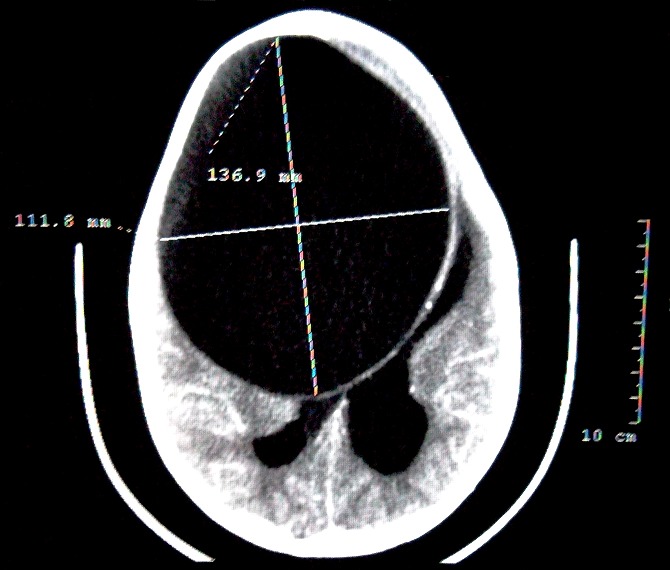
TDM cérébrale en coupe axiale montrant un énorme kyste hydatique fronto- pariétale

L'examen a trouvé une patiente comateuse avec un score de Glasgow à 4, apyrétique avec macrocranie et mydriase bilatérale réactive. Après mise en condition et intubation de la patiente, une TDM cérébrale a objectivé une énorme image arrondie hypodense fronto-pariétale d'environ 13,9 sur 11,6 cm faisant suspecter en premier lieu un kyste hydatique (Figure 1). L'échographie abdominale et la radiographie thoracique n'ont pas objectivé d'autres localisations.

**Figure 2:**
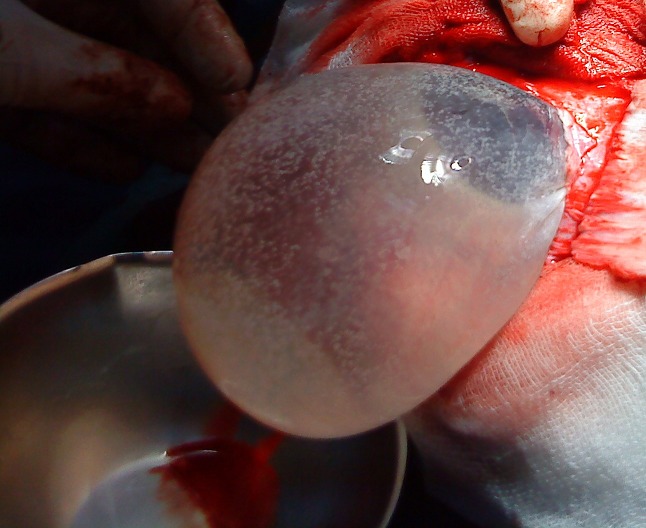
Aspect peroératoire du traitement du kyste hydatique cérébral

La patiente a été opérée le même jour avec réalisation d'une énucléation du kyste par hydrodissection selon la technique d'Arana Iniguez (Figure 2). L'étude anatomopathologique a confirmé un kyste hydatique. La patiente aséjourné en réanimation pendant 2 jours, avec bonne évolution puis admise au service de chirurgie ou elle a bénéficiée de soins locaux et de kinésithérapie motrice. La patiente estsortie 15 jours après l'acte opératoire, la reprise de la marche et de la parole a été acquise progressivement.

**Figure 3:**
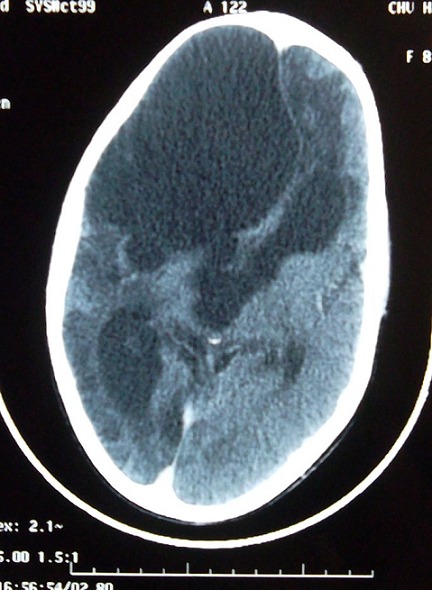
TDM cérébrale post opératoire montrant une cavité résiduelle

Un mois en postopératoire, la famille a constaté des troubles de comportement diagnostiqués comme étant un syndrome maniaque, la malade a bénéficié d'un scanner de control qui a montré une cavité résiduelle sans effet de masse (Figure 3), elle est suivie en psychiatrie avec bonne évolution. La patiente est suivie depuis deux ans avec bonne évolution.

## Discussion

La localisationcérébrale du kyste hydatique est rare représentant 1 à 2% des localisations chez l'homme [1, 2]. Elle est plusfréquente chez l'enfant et l'adulte jeune (50 à 70% des cas) et elle est le plus souvent solitaire [1, 4]. Ce parasite passe à travers le filtre hépatique et pulmonaire et rejoint le cerveau par la circulation systémique ce qui explique la rareté de cette localisation.

Les localisations cérébrales sont généralement hémisphériquessus-tentorielles et sous-corticales particulièrementdans le territoire de l'artère cérébrale moyenne au niveau du lobe pariétal [1, 2, 4]. De rares cas de localisations intraventriculaires et au niveau de la fosse postérieure ont été rapportés [1, 2]. La localisation cérébrale est dans 10% des cas associée à d'autres localisations viscérales notamment pulmonaireet hépatique [1]. Ces localisations doivent être systématiquement recherchées par la radiographie pulmonaire etl'échographie abdominale.

Le kyste hydatique cérébral se développe lentement de telle façon que la circulation collatérale va pouvoir pallier le risque d'ischémie [1] avec une vitesse de croissance d'environ 10 cm/an [1]. Cette lenteur du développement explique le fait qu'une hydatidose cérébrale peut atteindre un volume considérable avant de donner des manifestations cliniques [1] ce qui est le cas chez notre patiente, mais quand le kyste a pris un volume intolérable la détérioration neurologique est devenue plus rapide.

Le tableau clinique associe des signes neurologiques focaux associés ou non à une hypertension intra crânienne, rarement des troubles psychiatriques [1, 4]. L'examen physique peut montrer une augmentation du périmètre crânien chez le nourrisson et même chez le petit enfant ce qui est le cas de notre patiente, ce qui explique probablement l'évolution très progressive de la maladie. Le fond d"il montre souvent un ‘dème papillaire et une atrophie optique exceptionnellement [4]. La tomodensitométrie cérébrale constitue l'examen de référence. Elle visualise typiquement une masse kystique arrondie ou ovalaire, de volume variable, à contenu liquidien et à contours bien définis, ayant la densité du liquide céphalorachidien, et située en plein parenchyme cérébral. L'effet de masse sur les structures avoisinantes est important et une hydrocéphalie est possible par blocage des voies d'excrétion du liquide céphalorachidien. et pas de rehaussementaprès injection du produit de contraste [1–3], l"dème périlésionnel est exceptionnel [5]. La visualisation d'une membraneflottante est pathognomonique et les calcifications sont extrêmementrares, inférieures à 1% [1]. L'imagerie par résonance magnétique est rarement pratiquée et montre un hyposignal en T1 et un hypersignal enT2. Elle est meilleure dans la détection des kystes hydatiquescérébraux multiples et définit mieux les rapports de la lésionavec les structures avoisinantes ce qui aide à la planificationchirurgicale [2, 3, 6], de même l"dème périlésionnel est mieux visible par l'IRM. Le scanner est supérieur dans la détectiondes calcifications.

L'apport de la sérologie hydatique reste décevant par rapport aux autres localisations [2, 3]. La confirmation diagnostique est histologique. Le traitement est chirurgical basée sur la technique d'hydropulsion décrite par Arana Iniguez [1, 3], la deuxième technique possible est celle de la ponction'aspiration; elle est moins utilisée et réservée aux kystes ayant un risque important de rupture tels que les kystes duquatrième ventricule, les kystes du tronc cérébral et du thalamus [1]. Le traitement à base de benzimidazoles (albendazole et mébendazole) a été utilisé par certaines équipes en cas d'hydatidose récidivantes, disséminée, jugée inopérable ou rompue en peropératoire [1]. Les résultats du traitement médicamenteux deskystes hydatiques restent variables selon les séries, avec untaux de réponses allant de 43,5 à 80%.

Le pronostic est bon si le diagnostic est fait rapidement menant à un traitement précoce permettant d'éviter les séquelles neurologiques [7]. La prévention passe par les mesures d'abattage contrôlé des ovins.

## Conclusion

Le kyste hydatique cérébral est rare, d'évolution très lente, il peut prendre des dimensions importantes avant d'être symptomatique ce qui explique le retard diagnostic, le pronostic est bon après traitement chirurgical malgré la gravité des troubles neurologiques qui peuvent révéler la maladie.
